# FLNA is implicated in pulmonary neuroendocrine tumors aggressiveness and progression

**DOI:** 10.18632/oncotarget.20473

**Published:** 2017-08-24

**Authors:** Eleonora Vitali, Ilena Boemi, Lorenzo Rosso, Valeria Cambiaghi, Pierluigi Novellis, Giovanna Mantovani, Anna Spada, Marco Alloisio, Giulia Veronesi, Stefano Ferrero, Andrea G. Lania

**Affiliations:** ^1^ Laboratory of Cellular and Molecular Endocrinology, IRCCS Clinical and Research Institute Humanitas, Milan, Italy; ^2^ Thoracic Surgery and Lung Transplantation Unit, Fondazione IRCCS Ca' Granda Ospedale Maggiore Policlinico, Milan, Italy; ^3^ Humanitas Clinical and Research Center, Thoracic Surgery Division, Milan, Italy; ^4^ Fondazione IRCCS Ospedale Maggiore Policlinico, Endocrinology and Diabetology Unit, Department of Clinical Sciences and Community Health, University of Milan, Milan, Italy; ^5^ Department of Biomedical Sciences, Humanitas University, Milan, Italy; ^6^ Division of Pathology, Fondazione IRCCS Ca' Granda Ospedale Maggiore Policlinico, Milan, Italy; ^7^ Endocrinology Unit, Department of Biomedical Sciences, Humanitas University and Humanitas Research Hospital, Milan, Italy

**Keywords:** pulmonary neuroendocrine tumors, Filamin A, Rap1 GTPase, cell migration, cell proliferation

## Abstract

Pulmonary neuroendocrine tumors (PNTs) comprise different neoplasms, ranging from low grade carcinoids to the highly malignant small cell lung cancers. Several studies identified the cytoskeleton protein Filamin A (FLNA) as determinant in cancer progression and metastasis, but the role of FLNA in PNT aggressiveness and progression is still unknown.

We evaluated FLNA expression in PNTs with different grade of differentiation, the role of FLNA in cell proliferation, colony formation, angiogenesis, cell adhesion and migration in PNT cell line (H727 cells) and primary cultures and the possible interaction between FLNA and Rap1-GTPase. FLNA is highly expressed in PNTs with high malignant grade. FLNA silencing reduces cyclin D1 levels (-51±5, p<0.001) and cell proliferation in PNT cells (-37±4, p<0.05), colony formation and VEGF expression (-39±9%, p<0.01) in H727 cells.

FLNA and Rap1 co-localize in cellular protrusions and FLNA silencing up-regulates Rap1 expression (+73±18%, p<0.01).

Rap1 silencing prevents cell adhesion increase (+43%±18%, p<0.01) and cell migration decrease (-56±7%, p<0.01) induced by FLNA silencing, without affecting cell proliferation reduction. In conclusion, FLNA is implicated in PNT progression, in part through Rap1, thus providing a potential diagnostic and therapeutic target.

## INTRODUCTION

Pulmonary neuroendocrine tumors (PNTs) comprise a spectrum of neoplasms, ranging from low grade typical carcinoids via the intermediate grade atypical carcinoids to the highly malignant small cell lung cancers [1]. Since no large phase II/III trials for PNTs have been published, surgery remains the treatment of choice [2, 3]. Different studies clearly support a role of a widely-expressed cytoskeleton protein, Filamin-A (FLNA), in cancer progression and metastasis [4]. In fact, FLNA is overexpressed in multiple types of tumors, including prostate, breast, lung cancer, hemangiomas, colon cancer, melanoma, neuroblastoma, squamous cell carcinoma, hepatic cholangiocarcinoma, suggesting a possible correlation with FLNA and cancer aggressiveness [5, 6].

FLNA is crucial for cell shape modulation and motility: it crosslinks cortical actin filaments into a dynamic three-dimensional structure [7] and anchors actin filaments to cell-extracellular matrix adhesion sites. Moreover, FLNA is known to scaffold over 90 protein-binding partners, involved in receptor activation, cell migration and adhesion, cell proliferation, inflammation and tumorigenesis [8–10]. Increasing evidence demonstrated that FLNA is pivotal for regulation of matrix cytoskeleton signaling by binding to integrins, which are essential for maintaining directed cell migration and adhesion [6], and for coordination of GTPase signaling factors, leading the formation of lamellipodia and filopodia [11]. In this respect, Rap1 small GTPase is involved in the control of cell migration and adhesion by enhancing integrin-mediated cell–matrix attachment in several cell types [12].

In addition, Filamin-A (FLNA) physically interacts with HIF-1α [13], that regulates angiogenesis through upregulation of vascular endothelial growth factor (VEGF) [14, 15].

To date, the role of FLNA and the molecular mechanism involved in PNT aggressiveness and progression is still unknown. In order to establish whether FLNA is involved or not in this phenomenon we evaluated the correlation of FLNA expression patterns in different PNT stages, we assessed the role of this cytoskeleton protein in influencing cell mobility, cell proliferation, colony formation, angiogenesis, focusing on the potential interaction between FLNA and Rap1.

## RESULTS

### Filamin A expression correlates with different PNTs stages

**Figure 1:**
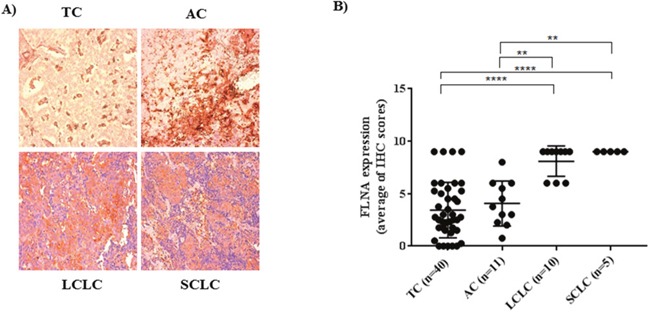
**(A)** Representative pictures of immunohistochemistry for FLNA in different PNTs (20X magnification). **(B)** Graph of FLNA expression obtained from the average of immunohistochemistry scores in different PNTs. FLNA immunoreactivity was graded taking into account both the percentage of positive cells (0–30%=1; 31–60% = 2; 61–100% = 3) and the staining intensity (0=absence of immunoreactivity; 1= weak; 2= medium intensity; and 3= strong reactivity). TC= typical carcinoid, AC= atypical carcinoid, LCLC=large cell lung carcinoma; SCLC= small cell lung carcinoma.

In order to study the possible relationship between FLNA and tumor aggressiveness, we evaluated FLNA expression in different PNT stages by immunohistochemistry using PNT samples, ranging from low grade typical carcinoids (TP) (n=40), atypical carcinoids (AT) (n=11) to the highly malignant large cell lung carcinoma (LCLC) (n=10) and small cell lung cancers (SCLC) (n=5). We found a correlation between clinical phenotype of different P-NETs and immunohistochemical score of FLNA. As shown in Figure 1A FLNA is expressed in the cytoplasm of PNT tissues and its expression being higher in PNTs with higher malignant grade. In particular, FLNA expression is significantly increased in LCLC and SCLC cells compared to TC and AC cells (one way ANOVA, followed by Bonferroni post hoc test, **** p<0.001, ** p<0.01) (Figure 1B). As shown in Table 1, FLNA expression is significantly correlated with age, gender, T and N staging (* p<0.05; ** p<0.01). The pathological findings of 65 PNTs included in the IHC study are described in the Supplementary Table 1.

**Table 1:** Associations between FLNA expression and clinicopathological features in PNTs

Factor	FLNA H scores (average)	P-value
**Age (years)**		**^a^
≤50	3,06547619	
>50	5,376539142	
**Gender**		**^a^
M	6,021481481	
F	4,032439024	
**T**		*^b^
T1	4,321891892	
T2	4,95	
T3	6,625	
T4	9	
**N**		*^b^
N0	4,229038462	
N1	5,125	
N2	7,178571429	

The association was identified with the appropriated statistical test, t-test or ANOVA when required.

F, female; M, male.

^a^ t-test; ^b^ ANOVA.

T, primary tumor; N, lymph node involvement.

### FLNA promotes PNTs cell proliferation and colony formation

**Figure 2:**
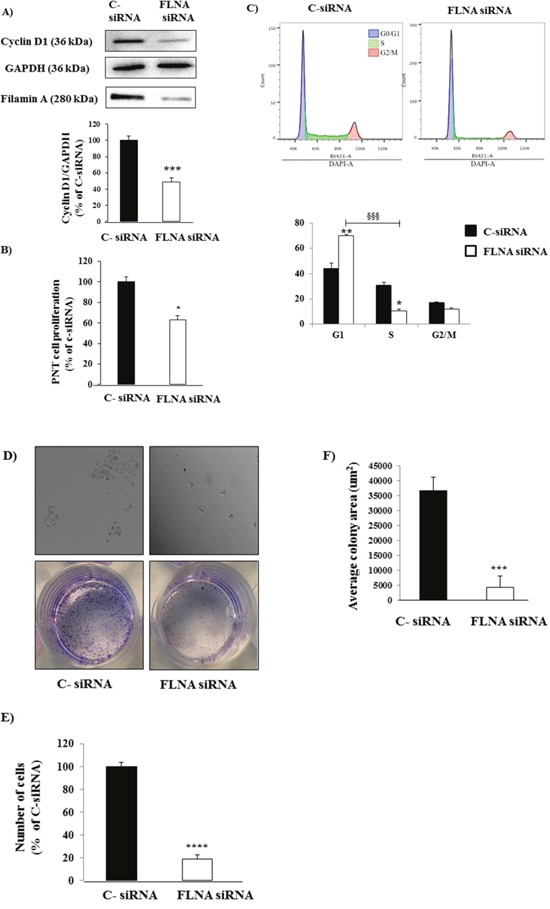
FLNA silencing reduced PNT cell proliferation and colony formation **(A)** Representative immunoblotting performed with antibodies raised against Cyclin D1. FLNA knock down induced a significant decrease in Cyclin D1 levels in primary PNT cells. The equal amount of protein was confirmed by stripping and reprobing with an anti-GAPDH antibody. The graph shows the quantification of Cyclin D1 normalized to GAPDH. Data represent mean ± SD of three independent experiments.*** =p<0.001 vs C- siRNA. **(B)** After FLNA silencing, we measured cell proliferation in primary PNTs cells incubated with BrdU for 24 h. Experiments were repeated 3 times and each determination was done in triplicate. Values represent mean (±SD) * =p<0.05 vs C- siRNA. **(C)** Cell cycle analysis by DNA content of H727 cells treated with C- siRNA or FLNA siRNA. H727 cell line were fixed and processed according to the DAPI labelling protocol listed on material and method. Single cells were gated via DAPI width and area signals to calculate G1, S phase, G2/M from a DAPI area histogram. Left panel represent H727 cells treated with C- siRNA, right panel represent H727 cells treated with FLNA siRNA. The graph shows the quantification of data from two independent experiments. Values represent mean (±SD) * =p<0.05, ** =p<0.01, §§§ =0.001. **(D)** FLNA silencing strongly decreased quantity of colonies. Microscopyimages (top panels) show H727 cells incubated with C-siRNA and FLNA siRNA after 7 days. Images of the cell colony assay stained with a solution containing 0.05% crystal violet (bottom panels). **(E)** To count numbers of cells, after 7 days H727 cells were incubated with cell quantification solution and absorbance was measured at 490 nm. Graph shows that FLNA silencing significantly decreased number of H727 cells with respect to C-siRNA cells. Values represent mean ±SD of 3 experiments. ****=p<0.0001 vs corresponding basal. **(F)** To measure colony surface area, 3-4 fields were randomly selected in each well. Average of colony area (μm2) was measured using the software ImageJ. ***=p<0.001 vs corresponding basal. Experiments were repeated at least 3 times. Statistical analysis was performed with t-test.

To further investigate the role of FLNA in tumor development and progression, we evaluated whether FLNA is involved in PNT cell proliferation. Thus, we silenced FLNA expression in PNTs to evaluate cyclin D1 expression. As shown, 80% decrease in FLNA levels significantly reduced cyclin D1 levels, with respect to PNT cells transfected with negative control (C- siRNA) (-51±5 p<0.001) (Figure 2A).

To confirm these data, we evaluated cell proliferation by BrdU incorporation during DNA synthesis. FLNA silencing significantly decreased cell proliferation in primary PNT cells (-37±4, p<0.05 vs C-siRNA (Figure 2B). These data suggest that FLNA promotes cell proliferation in PNTs.

To overcome the limitations due to the scarce availability and number of PNT cells, we used the human H727 cell line, as a model for typical carcinoids. To further investigate the promoting effects of FLNA in proliferation, we performed cell cycle analysis on H727 cells silenced for FLNA. As shown in Figure 2C FLNA knockdown significantly increased the percentage of H727 cells in the G1 phase and decreased in the S phase. Therefore, the cytostatic effect of FLNA silencing elicited by a decrease in cyclin D1 expression in PNTs cells is consistent with the inhibition of the cell cycle at the G1/S transition in H727 cell.

The tumorigenic properties of H727 cells transfected with C- siRNA or FLNA siRNA were compared by assessing their ability to grow *in vitro* and to form colonies.

As shown in Figure 2D, 7 days after plating, control H727 cells produced numerous colonies, whereas cells silenced for FLNA developed few and very small colonies. In addition, FLNA silencing significantly decreased the number of H727 cells (-80±3%, p<0.0001) and colony size (6.20 fold, p<0.001) with respect to negative control cells (Figure 2E, 2F).

### FLNA is involved in angiogenesis increase in H727 cells

**Figure 3:**
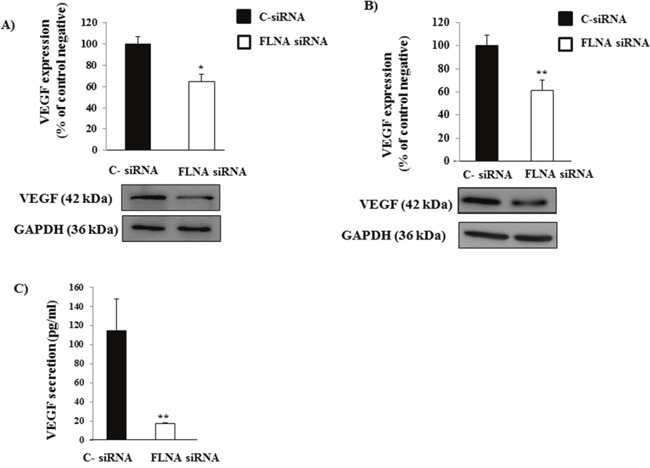
Effect of FLNA on VEGF expression and *in vitro* release in H727 cells **(A&B)** Representative immunoblot of VEGF demonstrates that FLNA silencing reduces VEGF expression in primary PNTs cells and H727 cells, respectively. The graph shows the ratio of VEGF/GAPDH normalized to C- siRNA. Experiments were repeated at least 2-3 times. *p<0.05, **p<0.01 vs C- siRNA. **(C)** ELISA analysis of VEGF release in supernatant by c-siRNA and FLNA siRNA H727 cells. All data are expressed as mean ± SD of three independent experiments. **=p <0.01, **=p<0.01 vs C- siRNA.

The VEGF pathway, implicated in angiogenesis, has been demonstrated to be over-expressed in PNTs [14], thus we evaluated FLNA implication in angiogenesis through links with vascular endothelial growth factor (VEGF). As shown in Figure 3A, 3B, VEGF expression decreased in primary PNT cells and H727 cells lacking FLNA (-36±7%, p<0.05 vs C-siRNA and -39±9%, p<0.01 vs C-siRNA, respectively). To confirm this result, we measured the levels of VEGF secreted by H727 cells transfected with C- or FLNA siRNA by ELISA. After 24 hours of culture VEGF release was drastically reduced in H727 cells transfected with FLNA siRNA compared to control, suggesting that FLNA silencing suppressed VEGF secretion. These data support the key role of FLNA in promoting angiogenesis.

### FLNA interacts with Rap1 in H727 cells

**Figure 4:**
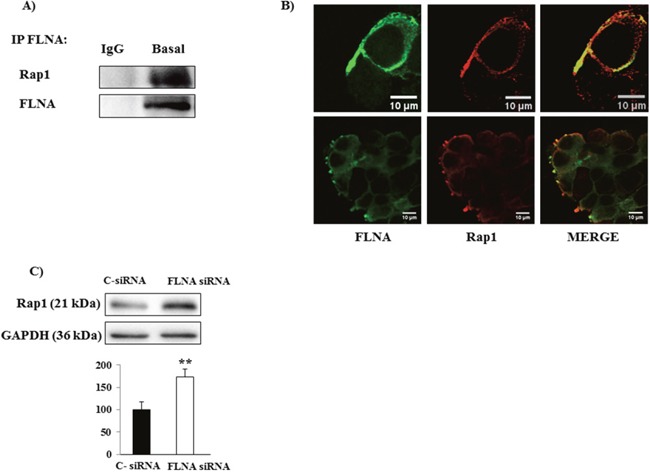
**(A)** Representative Immunoblot image shows thatFLNA interacts with Rap1. H727 lysate was immunoprecipitated with FLNA antibody or normal mouse IgG and the presence of Rap1 was detected using anti-Rap1 antibody. The presence of equal amounts of FLNA in the immunoprecipitates was confirmed by stripping and reprobing with anti-FLNA. As negative control, normal mouse IgG was used for the immunoprecipitation (IP). **(B)** Representative confocal microscopy images of fixed H727 cells stained for FLNA (green) and Rap1 (red). The results shown are representative images of three individual experiments. Scale bar 10 μm. **(C)** Representative immunoblots of Rap1 expression in H727 cells transfected with C-siRNA or FLNA siRNA. The graph shows the ratio of Rap1/GAPDH normalized to C-siRNA (mean value ±SD from 3 independent experiments). **=p<0.01 vs C- siRNA.

Since Rap1 small GTPase is involved in the control of cell migration and adhesion and it plays a key role in neuroendocrine tumors [16], we hypothesized that the effects of FLNA could be mediated, at least in part, by a modulation of Rap1 expression. To examine this hypothesis, we evaluated the possible interaction between FLNA and Rap1 by immunoprecipitation. As shown in Figure 4A, Rap1 interacts with FLNA in H727 cells. This result was confirmed by immunofluorescence analysis, in which FLNA co-localized with Rap1 and interestingly, they co-localized mainly in cellular protrusions (Figure 4B). Interestingly, FLNA silencing increased Rap1 expression levels in H727 cell with respect to control cells (+73±18%, p<0.01 vs C-siRNA) (Figure 4C), thus suggesting that FLNA is involved in the regulation of Rap1 expression.

### Implication of Rap1 in FLNA effect on cell proliferation, cell adhesion and cell migration

**Figure 5:**
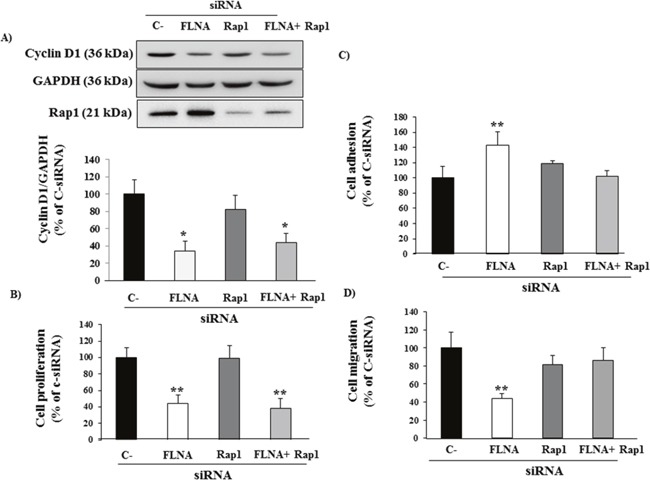
FLNA effect on cell proliferation, adhesion and cell migration and Rap1 involvement **(A)** FLNA siRNA-treated cells showed a strong decrease in Cyclin D1 expression and cell proliferation in H727 cells not via Rap1. H727 cells were transfected with control siRNA, FLNA siRNA alone or together with Rap1 siRNA. The graph shows the quantification of Cyclin D1 normalized to GAPDH. Data represent mean ± SD of three independent experiments. One-way ANOVA; Bonferroni post test * =p<0.05 vs C- siRNA. **(B)** The graph shows BrdU incorporation during DNA synthesis in H727 cells transfected with C-siRNA, FLNA siRNA alone or together with Rap1 siRNA. Data represent mean ± SD of three independent experiments. One-way ANOVA; Bonferroni post test **=p<0.01 vs C- siRNA. **(C)** H727 cells transfected with C-siRNA or FLNA siRNA alone or together with Rap1 siRNA were incubated in complete medium for 90 min at 37°C. Experiments carried out in H727 cells were repeated at least 4 times. The graph shows the quantification of adherent cells. Values represent mean (±SD). One-way ANOVA; Bonferroni post test **=p<0.01 vs C-siRNA. **(D)** H727 cells transfected with C-siRNA, FLNA siRNA alone or together with Rap1 were incubated for 24 h with serum-free medium. Migratory cells were quantified by fluorometric plate reader. Experiments were performed in triplicate 3 times. Values represent mean (±SD); One-way ANOVA; Bonferroni post test **=p<0.01 vs C-siRNA.

To characterize the mechanism by which FLNA affects cell proliferation in H727 cells, we co-transfected Rap1 siRNA and FLNA siRNA with the aim of blocking the over-expression of Rap1 induced by FLNA silencing. FLNA silencing strongly reduced cyclin D1 expression and cell proliferation in H727 cells (-61±2% p<0.05 vs C-siRNA and -56±11 p<0.01, respectively), confirming previous results in PNT cells. However, Rap1 knock down did not prevent the effects on cell proliferation of suppressing FLNA expression (Figure 5A, 5B). This result suggests that FLNA promotes cell proliferation in H727 cells, but not via Rap1.

Conversely, FLNA silencing increased cell adhesion (+43%±18%, p<0.01 vs C-siRNA) and decreased cell migration (-56±7%, p<0.01 vs C-siRNA). Interestingly, Rap1 silencing prevented the effects on adhesion and migration of suppressing FLNA expression in H727 cells (Figure 5C, 5D). These data suggest that FLNA effects on cell adhesion and migration are mediated by Rap1

## DISCUSSION

Cytoskeleton protein FLNA appears as a crucial element in PNTs cells as demonstrated in various types of cancers [5, 6], in which FLNA expression is correlated with the cancer metastatic potential.

In this study, we first found that FLNA is expressed in typical PNT samples and in the appropriate cellular model H727 cell line. In this respect, multiple studies detected FLNA over-expression in human glioblastomas [17], in pancreatic cancer [18], salivary gland adenoid cystic carcinoma [19], in peripheral cholangiocarcinomas [20] and many others.

We also showed that FLNA expression increased progressively with malignant grade, as reported in several studies, in which FLNA expression is correlated with cancer aggressiveness [21]. In this respect, circulating FLNA has been detected in plasma samples obtained from breast cancer patients using a monoclonal antibody, while no FLNA was found in plasma from normal subjects [22].

Next, the associations between FLNA expression and the clinicopathological features of the patients with PNT were investigated. Interestingly, FLNA expression is significantly correlated with clinical parameters, including age, gender, T and N staging.

Thus, the increase of FLNA expression in malignant PNTs, together with the correlation between its expression and additional histopathologic characteristics suggests that FLNA may be a useful tool for individualized therapeutic strategy and its expression may be considered as a potential diagnostic marker.

The knockdown of FLNA was associated with a decrease in metastasis and proliferation in different cancer cells, including human melanoma cells [23]. In this work, we report that FLNA promotes PNT cell proliferation and colony formation, crucial mechanisms involved in cancer progression. In particular, we demonstrated that the cytostatic effect of FLNA silencing is consistent with the inhibition of the cell cycle at the G1/S transition in PNT cells, corresponding to a decrease in cyclin D1 expression. These results confirmed the central role of FLNA in tumorigenesis.

Recently, the involvement of the cytoskeletal actin-binding proteins in angiogenesis has been suggested as a target for anti-neovascular cancer therapy *in vitro*. In particular, a positive relationship between FLNA and VEGF has been demonstrated in patients with lung cancer [14], suggesting that FLNA is implicated in the control of angiogenesis through links with VEGF. Interestingly, overexpression of VEGF, together with VEGF receptors, has been observed in PNTs, suggesting that an autocrine activation of VEGF pathway may be involved in PNT tumorigenesis and progression [2, 24]. Consistently with this observation, we demonstrated that FLNA silencing significantly decreased VEGF expression in PNT cells. Moreover, VEGF release was drastically reduced in H727 cells silenced for FLNA, suggesting that FLNA silencing also suppressed VEGF secretion. These data support the key role of FLNA in promoting angiogenesis.

Increasing evidence demonstrated that FLNA is essential for the regulation of cell migration and adhesion [25, 26]. In this respect, FLNA is required for podosome stabilization, extracellular matrix degradation and three-dimensional mesenchymal migration [27]. In our neuroendocrine model, we demonstrated that FLNA knock-down decreased cell migration and increased cell adhesion.

Due to its functions in the control of cell mobility, cell adhesion, cell proliferation, angiogenesis and colony formation, it is reasonable that FLNA may represent a biomarker for PNT diagnosis and prediction of clinical outcomes.

By virtue of its scaffolding function, FLNA also interacts with more than 90 functionally different proteins involved in tumorigenesis and metastasis [8, 10, 23]. In fact, FLNA anchors GTPase signaling proteins and coordinates their actin-remodeling activities, leading the formation of lamellipodia and filopodia [11]. In addition to the multiple proteins binding FLNA, we found that FLNA interacts with a novel downstream effector Rap1 small GTPase, and interestingly, these proteins colocalizes in cellular protrusions. In fact, Rap1 is involved in the control of cell migration and adhesion by enhancing integrin-mediated cell–matrix attachment in several cell types [12].

Mechanistically, we showed that FLNA down-regulates the expression of Rap1 in H727 cells. Since Rap1 is also involved in regulating cell adhesion and proliferation in PNT cells [16], we speculated that down-regulation of Rap1 expression by FLNA modulates these cellular activities, essential for cancer progression and metastasis development. Considering that FLNA knockdown increased Rap1 expression, we evaluated whether or not Rap1 over-expression silencing was able to abolish the effects of FLNA on cell proliferation, cell adhesion and migration. We concluded that FLNA increased cell migration and decreased cell adhesion by a Rap1-dependent pathway, but it increased cell proliferation via a Rap1-independent mechanism.

These results underline the crucial role of FLNA in cancer progression and aggressiveness and consequently its potential interest in PNTs therapy. In particular, the involvement of FLNA pathway in mediating PNT progression might not only provide valuable diagnostic and prognostic markers, but also novel therapeutic targets.

## MATERIALS AND METHODS

### Pulmonary neuroendocrine tumor cell cultures

The study was approved by the local ethics committee. Informed consent was obtained from all subjects involved in the study. Human neuroendocrine cells were obtained from 5 typical PNTs that were enzymatically dissociated in DMEM containing 2 mg/mL collagenase (Sigma–Aldrich CorporateHeadquarters St. Louis, MO) at 37°C for 2h. Dispersed cells were cultured in DMEM supplemented with 10% fetal bovine serum, 2 mM glutamine and antibiotics. Human typical bronchial carcinoid cell line H727 were purchased from ATCC in 2013. Cells were grown in RPMI 1640 medium supplemented with 10% FBS, 2 mM glutamine and antibiotics. H727 cell line was cultured at 37°C in 5% CO_2_ atmosphere. H727 were tested for mycoplasma contamination by using N-GARDE mycoplasma PCR reagent set (#EMK090020, Euroclone).

### Immunohistochemistry

Immunohistochemistry experiments were performed on sections from 66 PNTs retrieved from the archives of Pathology Unit of IRCCS Ospedale Maggiore Policlinico, Milan, Italy. After dewaxing in Bioclear and rehydrating in ethanol, the sections were pretreated in a water bath set to 98°C in 0.01 M citrate buffer for 25 minutes. FLNA antibody (MAB1678, Millipore, 1:600 diluition) was used, and antigen-antibody detection was performed with the MACH1 universal polymer detection kit (Biocare Medical). The evaluation of immunohistochemical staining was blind and FLNA immunoreactivities were graded according to an immunohistochemical score as previously described [28].

### FLNA and Rap1 silencing in pulmonary neuroendocrine cells

Gene silencing was performed in PNTs and H727 cells using species-specific human FLNA pre-designed siRNA (#4392420, Invitrogen) and Rap1 siRNA (#sc-36384 Santa Cruz), using Lipofectamine 2000 transfection reagent (#11668-019, Invitrogen) according to the manufacturer's instruction, for 72h and 48h of incubation, respectively.

In order to obtain the best efficiency of FLNA silencing three different human FLNA silencer select pre-designed siRNAs were tested.

Preliminary experiments to determine the optimal concentration of siRNAs and the kinetics of silencing of FLNA and Rap1 were performed. A negative control siRNA (C- siRNA) (AM4611, Invitrogen), a non-targeting sequence without significant homology to the sequence of human, mouse or rat transcripts, was used in each experiment. Western blotting was performed in each experiment to control the expression level of FLNA and Rap1 in silenced cells.

### Proliferation assay

Cell proliferation was assessed by colorimetric measurement of 5-bromo-2’-deoxyuridine (BrdU) incorporation during DNA synthesis in proliferating cells, according to the instruction of the manufacturer, as previously reported [29]. Briefly, after transfection with FLNA siRNA, Rap1 siRNA or Rap1 together with FLNA siRNAs, cells were incubated with complete medium for 24h hours and then with BrdU for 2h (cell lines) and 24h (primary cultures) to allow BrdU incorporation in newly synthesized cellular DNA. All experiments were repeated at least three times and each determination was done in triplicate.

### Colony formation

After FLNA silencing, H727 cells were seeded into 6-well plates and 96-well plates in triplicate at a density of 1000 cells or 250 cells, respectively, and incubated in a 37°C for 7 days. To visualize colonies, we fixed the cells for 10 min with 4% PFA and then we stained cell clones for 30 min with a solution containing 0.05% crystal violet, followed by water rinses to remove excess dye. The dishes were then photographed.

To obtain number of cells, we incubated cells with cell quantification solution, according to the manufacturer's protocol (#ECM570, Millipore), for 4 hours at 37°C and then, we measured absorbance at 490 nm. Images of H727 clones were acquired using brightfield microscopy with a 4X objective lens (Widefield IX53 Inverted, Olympus). Average of colony area is measured using the NIH software ImageJ.

### Cell cycle analysis

Cells were cultured in 6 well plates (6 × 10^5^ cells per well) with 1 ml of medium. After 72h of silencing fresh medium was added to perform the cell cycle analysis and left for 6 hours. Cells were harvested and washed twice in PBS 1X at 4°. Then cells were fix in 1ml of cold 80% ethanol adding drop wise to the pellet while vortexing and incubated at 4° for 30 min. After the cells were centrifuge at 2000rpm for 5 min to remove ethanol and then washed twice in PBS 1X at 4°. Finally the cells were stained for 30 minutes in DAPI solution (PBS, 0.01% TRITON X-100, 1 ug/ml DAPI-Invitrogen™) at RT and then DNA content was read at BD FACSymphony A5. The results were analyzed using FlowJo® software (FlowJo LLC).

### Immunoprecipitation of Rap1

H727 cells were lysed in lysis buffer in the presence of protease inhibitors. The homogenates were centrifuged for 10 min at 14,000 rpm at 4°C and the supernatant incubated with 2 μg of FLNA antibody (H00002316-M01, Abnova, 1:1000) and mixed overnight on a rotating wheel at 4°C.

Then 20 μl of resuspended volume of Protein A/G PLUS-Agarose beads (Santa Cruz) were added to the mix antibody-lysate and incubated at 4°C on a rotating device for 3 hours. Immunoprecipitates were collected by centrifugation at 2,500 rpm for 5 minutes at 4°C. The pellet was washed 5 times with lysis buffer, each time repeating the centrifugation step above. After the final wash, pellet was resuspended in SDS sample buffer containing reducing agents and analyzed by Western blotting, to visualize Rap1 (07-916, Millipore). The presence of equal amounts of receptor in the immunoprecipitates was confirmed by stripping and reprobing with anti-FLNA antibody. As a negative control, 2 μg of normal mouse IgG (#sc-2025, negative control, Santa Cruz) was used for the immunoprecipitation (IP).

### Fluorescence microscopy

H727 cells were fixed with 4% paraformaldehyde for 10 minutes, washed several times in PBS and treated with 0.1% Triton X-100 buffer. After blocking with 2% BSA and 5% NGS in PBS for 1 h, cells were incubated with anti-FLNA (MAB1678, 1:200, Millipore) and anti-Rap1 (1:50, 07-916, Millipore) antibodies and then stained with Alexa Fluor 488-conjugated secondary antibody and AlexaFluor 546-conjugated secondary (1:1000, Invitrogen, Carlsbad, CA) for 1h at room temperature.

Coverslips were mounted on glass slides with one drop of liquid mountant (ProLong® Gold Antifade Mountant, ThermoFisher). All images were collected using Olympus Fluoview FV1000 confocal microscope.

### Cyclin D1 and Rap1 western blotting

To analyze cyclin D1 expression level, H727 cells were treated with C- siRNA, FLNA siRNA, or Rap1 siRNA together with FLNA siRNA, then serum starved for 24h and incubated with complete medium for 6h at 37°C. Cells were then lysed in lysis buffer in the presence of protease inhibitors. Proteins were separated on SDS/polyacrylamide gels and transferred to a nitrocellulose filter. A 1:1000 dilution of anti-cyclin D1 was used as primary antibody (ENT1173, Elabscience, Bethesda, MD) and an antirabbit horseradish peroxidase-linked antibody was used as secondary antibody. To analyze Rap1 expression level, H727 cells were treated with C-siRNA and FLNA siRNA. Cells were then lysed in lysis buffer in the presence of protease inhibitors. Proteins were separated on SDS/polyacrylamide gelsand transferred to a nitrocellulose filter. The 1:1000 dilution of anti-Rap1 and an antirabbit horseradish peroxidase- linked antibody were used. GAPDH (AM4300, Ambion, 1:2000) was used as housekeeping.

### VEGF protein expression

To study VEGF expression, primary PNTs cell or H727 cells were transfected with FLNA siRNA or negative control siRNA for 72h and incubated with complete medium for 24 h at 37°C. Cells were then lysed in lysis buffer in the presence of protease inhibitors. Proteins were separated on SDS/polyacrylamide gels and transferred to a nitrocellulose filter. The 1:1000 dilution of anti-VEGF (ab46154, Abcam, UK) and an antirabbit horseradish peroxidase-linked antibody were used. GAPDH was used as housekeeping.

To analyse VEGF secretion, H727 cells were plated in 24-well tissue culture plates and incubated with FLNA siRNA or negative control siRNA for 72 h. After, fresh medium was added for 24h and then supernatants were collected. Media were analyzed by a commercially available sandwich human VEGF ELISA kit (DuoSet ELISA, R&D), according to the manufacturer's instructions. Assays were performed in triplicate for three independent experiments and the results were reported as picograms (pg) of VEGF per ml.

### Cell adhesion assay

H727 cells silenced for FLNA siRNA or Rap1 siRNA together with FLNA siRNA were plated onto a collagen type IV-coated 48-well plate and incubated with complete medium for 90 min at 37°C, as by manufacturer's protocol (CBA-061, Cell Biolabs INC). Briefly, nonadherent cells were removed by gently washing plates 4-5 times with PBS, adherent cells were lysed with lysis buffer and subsequently detected with CyQuant® GR Dye (Cell Biolabs INC). Finally, each extracted sample was quantified by measuring fluorescence with a fluorescence plate reader at 480 nm/520 nm. The experiment was performed four times in quadruplicate.

### Cell migration assay

H727 cells, transfected with negative control siRNA, FLNA siRNA or Rap1 together with FLNA siRNAs were plated in polycarbonate membrane plate (MDCBA-106, Cell Biolabs INC) in serum-free medium and placed into the feeder tray, containing chemoattractant solution, according to the instruction of the manufacturer. After 24 hours of incubation at 37°C, migratory cells were first dissociated from the membrane, then stained and quantified after extraction using a fluorometric plate reader.

### Statistical analysis

The results were expressed as the mean ± SD. A paired two-tailed Student's test was used to detect the significance between two series of data. P<0.05 was accepted as statistically significant.

One-way ANOVA analysis was used to compare three or more groups, followed by Bonferroni post-hoc test. Calculations were performed by GraphPad Prism 4.0 software (GraphPad Software, Inc., La Jolla, CA). P<0.05 was accepted as statistically significant.

## SUPPLEMENTARY MATERIALS TABLE





## Abbreviations

FLNA: filamin-A
PNT: pulmonary neuroendocrine tumor
HIF-1α: hypoxia-inducible factors-1α
VEGF: vascular endothelial growth factor
siRNA: small interfering RNA
BrdU: 5-bromo-2’-deoxyuridine
IP: immunoprecipitation
GAPDH: Glyceraldehyde-3-Phosphate Dehydrogenase
