# Supplementary material for: FLNA is implicated in pulmonary neuroendocrine tumors aggressiveness and progression
Source: Oncotarget. 2017 Aug 24;8(44):77330–40. doi: 10.18632/oncotarget.20473 (PMC5652783; doi:10.18632/oncotarget.20473)
Supplement: Supplementary file 1 [file oncotarget-08-77330-s001.pdf]

FLNA is implicated in pulmonary neuroendocrine tumors aggressiveness and progression

SUPPLEMENTARY MATERIALS

Supplementary Table 1: Pathological findings of PNTS included in the IHC study

See Supplementary File 1
