# Supplementary material for: FLNA is implicated in pulmonary neuroendocrine tumors aggressiveness and progression
Source: Oncotarget. 2017 Aug 24;8(44):77330–40. doi: 10.18632/oncotarget.20473 (PMC5652783; doi:10.18632/oncotarget.20473)
Supplement: Supplementary file 2 [file oncotarget-08-77330-s002.doc]

| **Case number**  **Supplementary Table 1: Pathological findings of PNTs included in the IHC study** | **Types of PNTs** | **Age** | **Gender** | **TNM** |
| --- | --- | --- | --- | --- |
| 1 | TC | 48 | M | T2N0 |
| 2 | TC | 48 | F | T2N0 |
| 3 | TC | 53 | F | T1N0 |
| 4 | TC | 63 | F | T2N0 |
| 5 | TC | 20 | F | T2N0 |
| 6 | TC | 68 | M | T1N0 |
| 7 | TC | 74 | F | T1N0 |
| 8 | TC | 77 | F | T2N0 |
| 9 | TC | 57 | M | T2N0 |
| 10 | TC | 74 | F | T1N0 |
| 11 | TC | 47 | M | T2N0 |
| 12 | TC | 79 | F | T1N0 |
| 13 | TC | 30 | F | T1N0 |
| 14 | TC | 57 | F | T1N0 |
| 15 | TC | 57 | F | T1N0 |
| 16 | TC | 24 | F | T2N0 |
| 17 | TC | 61 | F | T1N0 |
| 18 | TC | 51 | F | T1N0 |
| 19 | TC | 31 | M | T1N0 |
| 20 | TC | 80 | F | T1N0 |
| 21 | TC | 67 | F | T1N0 |
| 22 | TC | 45 | M | T1N0 |
| 23 | TC | 58 | F | T1N0 |
| 24 | TC | 51 | F | T2N0 |
| 25 | TC | 35 | F | T1N0 |
| 26 | TC | 62 | F | T2N0 |
| 27 | TC | 32 | F | T1N0 |
| 28 | TC | 56 | F | T1N0 |
| 29 | TC | 29 | M | T1N0 |
| 30 | TC | 69 | F | T3N1 |
| 31 | TC | 67 | F | T1N0 |
| 32 | TC | 78 | F | T2N0 |
| 33 | TC | 82 | F | T1N0 |
| 34 | TC | 60 | F | T1N0 |
| 35 | TC | 78 | F | T2N0 |
| 36 | TC | 50 | F | T1N0 |
| 37 | TC | 72 | F | T1N0 |
| 38 | TC | 73 | M | T2N0 |
| 39 | TC | 67 | F | T1N0 |
| 40 | AT | 38 | M | T3N1 |
| 41 | AT | 80 | M | T2N1 |
| 42 | AT | 75 | F | T2N2 |
| 43 | AT | 67 | F | T1N0 |
| 44 | AT | 71 | M | T1N0 |
| 45 | AT | 53 | M | T1N0 |
| 46 | AT | 39 | F | T1N0 |
| 47 | AT | 59 | M | T1N0 |
| 48 | AT | 18 | F | T2N0 |
| 49 | AT | 36 | F | T1N2 |
| 50 | AT | 63 | M | T1N0 |
| 51 | LCLC | 54 | F | T2N2 |
| 52 | LCLC | 54 | M | T2N0 |
| 53 | LCLC | 64 | M | T2N1 |
| 54 | LCLC | 55 | M | T2 |
| 55 | LCLC | 42 | M | T2N2 |
| 56 | LCLC | 66 | M | T1N0 |
| 57 | LCLC | 74 | M | T4N0 |
| 58 | LCLC | 75 | M | T1N1 |
| 59 | LCLC | 77 | M | T2N2 |
| 60 | LCLC | 56 | F | T1N0 |
| 61 | SCLC | 54 | M | T3N2 |
| 62 | SCLC | 58 | F | T3N2 |
| 63 | SCLC | 60 | F | T1N0 |
| 64 | SCLC | 52 | M | T4 |
| 65 | SCLC | 72 | F | T3 |
